# Supplementary figures and images for: The Analysis of Transcriptomes and Microorganisms Reveals Differences between the Intestinal Segments of Guinea Pigs
Source: Animals (Basel). 2022 Oct 25;12(21):2925. doi: 10.3390/ani12212925 (PMC9658385; doi:10.3390/ani12212925)

**A****PSI**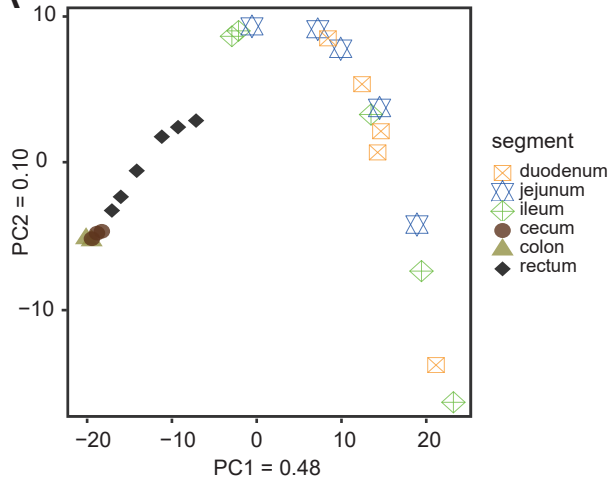**B****observed features**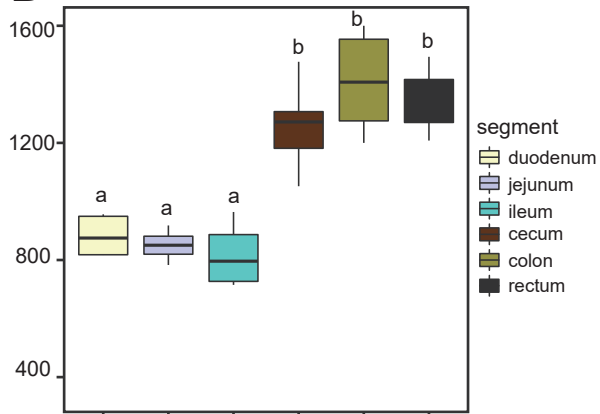

Supplement: Supplementary file 1 [file animals-12-02925-s001.zip › Figure S1.pdf]
